# Supplementary material for: Unraveling the dynamics of dengue in Metahara town, East Shewa, Oromia, Ethiopia, 2023
Source: PLoS Negl Trop Dis. 2025 Mar 17;19(3):e0012908. doi: 10.1371/journal.pntd.0012908 (PMC11957386; doi:10.1371/journal.pntd.0012908)
Supplement: S1 Text — (DOCX) [file pntd.0012908.s001.docx]

**S1: Consent Form**

I have read the above information, or it has been read to me. I was allowed to ask questions and the questions that I have asked have been answered to my satisfaction. I understand that I have the right to withdraw from the study at any time and can skip questions that I am not comfortable to respond. Therefore, after I understand the level of my satisfaction I agree and consent voluntarily to participate in this study.

Signature of volunteer: **__________________** Date: __________________________

Signature of Data collector: ______________________ Date: __________________
