# Supplementary material for: Unraveling the dynamics of dengue in Metahara town, East Shewa, Oromia, Ethiopia, 2023
Source: PLoS Negl Trop Dis. 2025 Mar 17;19(3):e0012908. doi: 10.1371/journal.pntd.0012908 (PMC11957386; doi:10.1371/journal.pntd.0012908)
Supplement: S2 Text — (DOCX) [file pntd.0012908.s002.docx]

**Dengue Outbreak Investigation Tool**

**Woreda profile**

Name of woreda, Zone and Region respectivelyTotal population of the woreda in 2023Distance of the woreda from AA and zonal town *KM*Name of InterviewerPatient response to participate

Yes... continueNo... Stop

**Part I: Socio-demographic characteristics**

1. Patient ID2. Sex

MaleFemale

3. Age 4. Address *Kebele, Woreda*

5. Marital Status

NASingleMarriedWidowedDivorced

6. Ethnicity

OromoAffarSumaleAmharaTigreOther

7. Educational status

NAUnable to read and writePrimarySecondaryCollege and above

8. Occupation

NAStudentFarmerMarchentDaily laborerGov/NGO employee other

**Part II: Clinical presentation**

9. Case status

CaseControl

10. Date of Onset*DDMMYYYY*

11. Signs and symptoms

FeverHeadRashChillNasal Bleeding/bleeding from any part of the body abdominal pain ever muscle and joint painRestlessness/lethargy nausea/vomitingOther

Please, specify if other12.The date seen at the health facility may

13. Treatment given

AntibioticAntipyreticAnti malariaAntiviralOther

Please, specify if other14. Is the sample taken for Dengue?

YesNo

15. Date sample collected?

16. Positive for Dengue?

YesNo

17. For which serotypes?

Type 1Type 2Type 3Type 4

18. Patient outcome

LiveDied

**Part III: Risk Factors Assessment**

19. Have you ever been infected by Dengue?

YesNo

20. Do you have LLINs?

YesNo

21. Is there any water-holding container in/around the house?

YesNo

please, specify if other22.Is there any stagnant water around your village?

YesNo

23. Is your house sprayed?

YesNo

24. Is there any river around your village?

YesNo

25. Is there any person diseased in your family?

YesNo

26. Did you have close contact with a person with the same complaint within the last 1 to 2 weeks?

YesNo

27. Did you have a travel history within the last two weeks?

YesNo

28. Do you use mosquito repellents on your skin?

YesNo

29. Do you use mosquito repellant in your house?

YesNo

30. What kind of clothes do you usually wear?

Short and T-shirtsTrousers/ body full dress

**Part IV: Knowledge Assessment**

31. Do you hear about Dengue?

YesNo

32. Is it transmittable disease?

YesNo

33. How it could be transmitted?

Mosquitos bite through air dropletHouse fly others

please, specify if other34. At which time mosquito bites people?

NightDayDay and night unknown

35. Does water required for mosquitoes to breed?

YesNo

36. What are the symptoms of Dengue?

Head acheFeverBleedingMuscle or abdominal pain nausea and vomiting other please, specify if other.
