# Supplementary material for: Unraveling the dynamics of dengue in Metahara town, East Shewa, Oromia, Ethiopia, 2023
Source: PLoS Negl Trop Dis. 2025 Mar 17;19(3):e0012908. doi: 10.1371/journal.pntd.0012908 (PMC11957386; doi:10.1371/journal.pntd.0012908)
Supplement: Attachment — Submitted filename: PNTD-D-24-00552_Reviewer_comments_23_05_2024.docx [file pntd.0012908.s003.docx]

The following issues need to be revised/improved on the manuscript

**Methods**

1. Improve the clarity of Figure 1 (Map of Metahara town) by following PLOS-NTD image standards and formats, such as PNG, TIFF, or PDF with a resolution of 300 dpi.
2. The description of sampling procedures is lacking details on how blood samples were collected from cases and control groups and transported to the testing laboratory.
3. For case definitions: Epidemiological case: Why were positive malaria test cases excluded? The presence of malaria does not rule out concurrent dengue fever infection, particularly during outbreaks or in endemic tropical settings where malaria-dengue co-infection is common. For example, in Sudan, malaria-dengue co-infections have been recorded up to >10% in some areas (Ahmed et al., 2021) Source. Confirmed case: The definition needs to be revised to follow the WHO dengue guidelines for diagnosis, treatment, prevention, and control (WHO, 2009). IgM antibodies can persist in the blood for up to three months, making it difficult to differentiate between active infection and recent exposures. According to WHO, a confirmed case is RT-PCR+ within 2-7 days after the onset of fever or IgM+ seroconversion in paired sera (acute sample tested against convalescent sample collected after 7 days). The use of RT-PCR in the study should be clearly included in the confirmed case definitions. IgM+ alone does not meet the criteria for a confirmed dengue case as per WHO guidelines.
4. The description of laboratory testing of samples is missing. Add an explanation of laboratory testing of samples using RDT and later using the RT-PCR method.
5. The statistical analysis was correct for the intended study objectives.
6. Ethical clearance was properly adhered to in the study.

**Results**

1. The analysis provided agrees with the analysis plan.
2. The results are not clearly presented.
3. The tables should be formatted according to standard publication rules, e.g., tables should have no internal lines except for headers.
4. I recommend recategorizing the age groups to indicate extreme age groups, e.g., 0-4 yrs, 5-14 yrs, 15-24, 25-34, 35-44, 45-54, 55-64, 65+. This is important in the context of dengue immune response and severe disease development, which differ between these age groups, with higher risk observed in children and adults over 60. Immunity for preschool and school children may differ due to varying levels of exposure to dengue infection, with higher risk in the older group (>50) due to enhanced immunity following secondary infections with different dengue types that lead to cross-reactive immune complexes and release of inflammatory agents. These age groups should be consistent in Table 1 and Table 2.
5. Section 3.2.3, "Factors associated with dengue fever outbreak," should be revised to "Risk factors associated with dengue fever outbreak."
6. All figures lack captions (subtitles) and explanations. They should be well-labeled, e.g., Figure A, B, C, etc., and include the source and date, e.g., Source: John et al., 2014.
7. There are many grammatical errors and typos in this section that need to be revised.
8. All scientific names should be correctly written in italics, e.g., Aedes aegypti.

**Conclusions**

1. The conclusions are supported by the data presented; however, there is significant redundant information, particularly repeated results and discussion points. For instance, results such as “more cases were reported in males than females with increased severity, admission rate, and case fatality rate” and “the presence of water containers in or around households and not using LLINs during the day were significant risk factors” are repeated without adequate implications being provided. These statements are part of the results rather than the conclusions.
2. The author should synthesize these results to provide detailed interpretations and implications. Specifically, the author should explain how these findings can inform public health interventions in Metahara town, particularly in terms of implementing or strengthening vector control, dengue surveillance, and preparedness against future outbreaks, as mentioned in the introduction section.
3. The recommendation section should be removed to adhere to the standard "IMRaDC" format; Introduction (the study's purpose), Methods (how the study was conducted and the justification for the chosen methods), Results (what was found), Discussion (the meaning of the findings), and Conclusions (implications and lessons learned from the findings).
4. The limitations of the analysis have not been discussed. Additionally, the authors have not sufficiently explained how the data advances understanding of the topic. More synthesis and interpretation are required to address this.

**Editorial and Data presentation**

A minor revision is recommended to enhance the quality of data presentation and general manuscript formatting. The paper is worthy of publication once the suggested comments have been appropriately addressed by the authors.

**Summary and General Comments**

Ethiopia has experienced a surge in dengue virus infections since 2019, highlighting the need for research to inform public health interventions. The current study is original research focusing on Metahara town in Ethiopia, providing valuable information for public health decision-makers to improve preventive strategies and save lives. No further experiments are required in the current format, but minor revisions to address all comments are needed. Additionally, I recommend revising the manuscript title to "Dengue Fever Outbreak Investigation and Associated Risk Factors in Metahara Town, Ethiopia, 2023."
