# Supplementary material for: Unraveling the dynamics of dengue in Metahara town, East Shewa, Oromia, Ethiopia, 2023
Source: PLoS Negl Trop Dis. 2025 Mar 17;19(3):e0012908. doi: 10.1371/journal.pntd.0012908 (PMC11957386; doi:10.1371/journal.pntd.0012908)
Supplement: Attachment — Submitted filename: Bikila_DF_Response_to_Reviewers.docx [file pntd.0012908.s004.docx]

**Point-by-point responses to reviewer’s and editor's comments**

**Journal**: PLOS Neglected Tropical Diseases

(PNTD-D-24-00552) - [EMID: be8108a824f88701]

Dear Editor-in-Chief,

We thank you and the reviewers for your appraisal and constructive comments on our manuscript titled "**Unraveling the dynamics of Dengue in Metahara town, East Shewa, Oromia, Ethiopia, 2023**". We have provided a detailed response to each reviewer’s comment. In addition, the clean (unmarked) and marked-up versions of the manuscript were highlighted in yellow to show changes made.

We look forward to hearing your positive feedback and consideration of the revised manuscript for publication.

Sincerely,

Mr. Bikila Negesa Gobena, Principal Investigator

A. **Comments from AE**

- There are a large number of grammatical errors in the manuscript, some are listed by the reviewers, please have the manuscript independently proofed.
  For example, "Dengue patients those did not use LLINs during day time were 9.07 time more likely to had odd of developing dengue (AOR= 9.07, 95% CI: 2.05-40.06)"

*Response: Thank you for your comment. We accepted the comment and made revisions to the manuscript as “Dengue patients who did not use Long-Lasting Insecticide Nets (LLINs) (LLINs) during daytime were 9.07 times more likely risk of developing Dengue (AOR= 9.07, 95% CI: 2.05-40.06) compared to those who did not use it*”

- Second example, "Twenty liter of abate® chemical was given for distributed for town health office"

*Response: We accepted the comment and corrected as “*From August 5 to 10/2023, Abet Chemical of 20 liters used as larvicidal was distributed and sprayed to the identified *Aedes aegpti* mosquitos breeding sites”

Third example, the "regional state, Ethiopia the "

*Response: Thank you for your comment. We accepted the comment, and revisions were made to the commented part in the revised manuscript as “*In July 2023, a suspected outbreak of Dengue was reported in Metahara town, found in Oromia regional state, Ethiopia. This study aimed to confirm the existence of the dengue outbreak, identify the associated risk factors, describe the epidemiological characteristics, and undertake control measures.”

- There are many examples across the ms.

*Response: Thank you for your comment. We accepted the comment, and revisions were made to the grammatical errors throughout the entire document in the revised manuscript*

- The Excel graphs are below publication standard, for example Fig. 2 the title of the x-axis mixes with the labels.

*Response: Thank you for your comment and here below are the clarification points.*

“Figure 2 was narrowed beyond the standard, resulting in the x-axis mixing with the labels. After we expand it to the standard, the problem is already solved.”

- The tables are not publication format and the formats keep changing between tables

*Response: Thank you for your comment and here below are the clarification points.*

“Table format keeps changing between tables is removed"

- CAR and ASAR need defining (Table 1)

*Response: Thank you for your comment and here below are the clarification points.*

*Corrected as “*CAR to Crude attack rate and ASAR to Age-specific attack rate*”*

- Table 2, this is fine for the appendix and some demographic characteristics. Age distribution needs plotting between total population and case control to assess the distributions.

*Response: Thank you for your comment and here below are the clarification points.*

*”* *We examined these points in great detail and we were fully appreciative of the comments provided by AE, as they can assist us in comprehending the potential confounding variables and making necessary adjustments to their analysis. Nonetheless, in our research, we employed an unmatched case-control study design, which yielded a null effect in the age category, indicating that during the current Dengue outbreak, there are no significant distinctions in the outcomes between the control and case cohorts. This phenomenon arises when the age category does not exert a noteworthy influence on the outcome variable under investigation. Put differently, the age category does not impact the correlation between exposure and the study findings.*”

- Table 2, I suggest removing the percentages - it becomes difficult to read. It is debacle whether other demographics are better plotted particularly Occupation and Education.

*Response: Thank you for your comment it is revised as “We removed the percentages part as per your recommendation” corrected as*

| Variables Categories | | Case n=50 | Control n=100 | Total N=150 |
| --- | --- | --- | --- | --- |
| Sex | Female | 25 | 41 | 66 |
|  | Male | 25 | 59 | 84 |
| Marital Status | Single | 11 | 26 | 37 |
|  | Married | 39 | 74 | 113 |
| Educational status | Not applicable | 2 | 0 | 2 |
|  | Unable to read and write | 10 | 5 | 15 |
|  | Primary | 14 | 8 | 22 |
|  | Secondary | 11 | 62 | 73 |
|  | College and above | 13 | 25 | 38 |
| Occupational status | Contractor | 4 | 2 | 6 |
|  | Daily laborer | 12 | 21 | 33 |
|  | GO/NGO employee | 9 | 17 | 26 |
|  | Manufacturer | 4 | 2 | 6 |
|  | Merchant | 14 | 39 | 53 |
|  | Not applicable | 2 | 0 | 2 |
|  | Student | 5 | 19 | 24 |
| Age category | <=4 | 1 | 0 | 1 |
|  | 5-14 | 3 | 0 | 3 |
|  | 15-34 | 19 | 62 | 81 |
|  | 35-44 | 10 | 19 | 29 |
|  | >=45 | 17 | 19 | 36 |

- It appears adjusted odds ratio analysis did not include, gender marital status, education, occupation and age. Why not?

*Response: Thank you for your comment and here below are the clarification points.*

*“We displayed here the independent variables those fulfill the candidate variable criteria. Gender, marital status, occupation, education and age were not candidate for the final model (Multivariate analysis). As you said, it was better if we showed it, but we made it to shortened”*

- LLIN AOR this makes absolute sense, but there is very little background information on a key result. The Introduction and Conclusion needs to go into substantial more detail about LLIN day time use. How does this work? How is it linked to profession? How do people practical use an insecticide net during the day? How do you conclusions link to future improved disease prevention?

*Response: Thank you for your comment and here below are the clarification points.*

*“Yes, it was the ideal point! We included all accordingly, in the introduction and conclusion parts with very details.”*

- The entire discussion should be expanded.

*Response: Thank you for your comment. We accepted the comment, and revision was made in* the *revised manuscript.*

- * Please re-write your Abstract: a) to address a large number of typos therein, b) help us understand the result in an epidemiological context (e.g. insecticide nets) and b) generally the results of AOR and other tests are presented in words in an abstract state, e.g. whether a given result is significant (in words). For example AOR is never defined in the Abstract, so it's really confusing what it means for anyone outside epidemiology. d) the conclusion needs strengthening, e) none of results are discussed in the conclusion, for example issue recommendations for control.
- * Abstract and analysis "Male and age between 15-44 years old were more affected. " needs statically verifying and stating in writing.
  I will be reviewing all statistics on resubmission.

*Response: Thank you for your comment and here below are the clarification points.*

- *“*We used age category, sex, occupation, marital status, and education only for descriptive purposes because they had no significant association with the outcome of interest. So, we agreed to remove them from the abstract*”*

*Responses: Thank very much for comments. We revised the abstract part as “****Abstract***

***Background:*** *Dengue is a mosquito-borne viral disease that has been increasingly reported in Ethiopia in recent years. In July 2023, a suspected outbreak of Dengue was reported in Metahara town, found in Oromia regional state, Ethiopia. This study aimed to confirm the existence of the Dengue outbreak, identify the associated risk factors, describe the epidemiological characteristics, and undertake control measures.*

***Methods****: A descriptive study was followed by an unmatched case-control study of 1:2 case-to-controls. Data on Dengue outbreak, including socio-demographic, geographical distribution, clinical presentation, and number of cases, was gathered from line lists, laboratory test results, environmental observation, home visits, and entomological examination. The study used the line list as a sample frame when selecting 50 cases using simple random sampling and 100 controls from the same block as cases purposively, with community-based data collection. Finally, community based face-to-face interviews were conducted with a total of 150 study participants. The collected data using Kobo Collect was cleaned, checked, and expertly converted to SPSS version 26 for analysis, while Microsoft Excel 2013 was used for descriptive. The study used a binary logistic regression model, identifying variables with p-values ≤ 0.25 in bivariate analysis and significant for the Chi-square test for the final model. The strength of the association was measured using the crude odd ratio and the adjusted odd ratio. In multivariate analysis, variables with p-values ≤ 0.05, adjusted odds ratio, and 95% CI are used to declare statistical significance. Hosmer-Lemeshow was used to test the model's goodness of fit*

***Results:*** *The investigation revealed 342 confirmed and epidemiologically linked Dengue cases from July 28 to September 5/2024, with an attack rate of 7.1 per 1,000 populations, 7% hemorrhagic fever cases, and a fatality rate of.88%. Individuals who were not utilizing long-lasting insecticide nets during the day were 9.07 times more likely to be associated with a risk of Dengue outbreak, while households with open water containers in or around their home had a 4.99 times higher risk. Respondents without information about the disease were 25.2 times more likely to have the infection, and wearing long-sleeved clothing was 75% more protective than wearing short sleeves.*

***Conclusion****: A Dengue outbreak in Metahara town, Ethiopia, has been confirmed, with 342 cases. Risk factors include not using long-lasting nets, open water storage, and lack of information. Long-sleeved clothing is protective. Preventing future outbreaks requires data integration, evidence-based vector management, community engagement, and multi-sectorial coordination.*

***Keywords****: Dengue Outbreak, Metahara town, Ethiopia, 2023*”

B. **Technical Check requests for your submission**
1. We note that your MANUSCRIPT files are duplicated on your submission. Please remove the manuscript with .pdf format leaving only the manuscript with .docx format.

*Responses: Yes, it was because of technical error, but now it is resolved*

2. Please upload all main figures as separate Figure files in .tif or .eps format. For more information about how to convert and format your figure files please see our guidelines: LINK <https://journals.plos.org/plosntds/s/figures>

*Response: We accept your comments and we put figures separately using the above link.*

3. We have noticed that you have cited Table  3 in the manuscript file but there is no corresponding table in the manuscript.  Please amend your manuscript to include this table noting that tables should not be uploaded as individual files.

*Response: We include table 3 in manuscript file.*

4. We notice that your supplementary information is included in the manuscript file. Please remove them and upload them with the file type 'Supporting Information'. Please ensure that each Supporting Information file has a legend listed in the manuscript after the references list

*Response: Thank you very much, we arranged it in the revised manuscript by uploaded the supporting materials in individual files after we legend it in manuscript next to reference.*

5. Please remove the pictures (3.3.1 Coordination,3.3.2 Surveillance activities,3.3.3 Vectors Control Activities,3.3.4 Diagnosis and Case Management) from the manuscript file and re-upload them as individual FIGURE files and also ensure that  all Figure files have corresponding CITATIONS within the manuscript. Please re-label and number them accordingly as Figure 1, Figure 2 etc	.
   NOTE: We don't publish any graphics embedded in articles - they all need to be labeled as figures

*Responses: Thank you again! We created file for figure and uploaded as individual folder*.

6. Potential Copyright Issues:
   1. [3.3.1 Coordination,3.3.2 Surveillance activities,3.3.3 Vectors Control Activities, 3.3.4 Diagnosis and Case Management ]: Please confirm (a) that you are the photographer; or (b) provide written permission from the photographer to publish the photo(s) under our CC BY 4.0 license.

*Responses: We accept you comment and we removed it in the revised manuscript.*

2) [3.3.1 Coordination,3.3.2 Surveillance activities,3.3.3 Vectors Control Activities, 3.3.4 Diagnosis and Case Management ] includes an image of an identifiable person. Please provide written confirmation or release forms, signed by the subject(s) (or their parent/legally authorized guardian), giving permission to be photographed and to have their images published under our CC BY 4.0 license. Otherwise, we kindly request that you remove the photograph.

*Responses: We accept you comment and we removed it in the revised manuscript.*

3) [3.3.4 Diagnosis and Case Management]: Please confirm whether you drew the images / clip-art within the figure panels by hand. If you did not draw the images, please provide (a) a link to the source of the images or icons and their license / terms of use; or (b) written permission from the copyright holder to publish the images or icons under our CC BY 4.0 license. Alternatively, you may replace the images with open source alternatives. See these open source resources you may use to replace images / clip-art:
- [https://commons.wikimedia.org](https://commons.wikimedia.org/)
- <https://openclipart.org/>

*Responses: We accept you comment and we removed it in the revised manuscript.*

4) [3.3.4 Diagnosis and Case Management] contains branding/a logo. We are not permitted to publish this under our CC BY 4.0 license, even with permission. We ask that you please remove or replace it.

*Responses: We accept you comment and we removed it in the revised manuscript*

5) [3.3.2 Surveillance activities,3.3.3 Vectors Control Activities] includes child photographs. Please provide explicit written consent to publish these images from the child's parent/guardian
NOTE: Please de-identify the faces with a black band across their eyes (or blurred) or provide written permission to use their images with the consent form

*Responses: We accept you comment and we removed it in the revised manuscript*

5) Figure [1]: please (a) provide a direct link to the base layer of the map (i.e., the country or region border shape) and ensure this is also included in the figure legend; and (b) provide a link to the terms of use / license information for the base layer image or shapefile. We cannot publish proprietary or copyrighted maps (e.g. Google Maps, Mapquest) and the terms of use for your map base layer must be compatible with our CC BY 4.0 license.

*Responses: Thank you very much for comments once again. We describe our responses as follow: Regarding the shape file of figure one: we have included the link of the source of the shape files in the main manuscript at ((https://data.humdata.org/dataset/cod-ab-eth). We used to locate the Ethiopia boundary from Central Statistical Authority 2019, which is available to use for the public good. We included the software developers for using freely accessible QGIS software. The research team compiled and organized the map for the purpose of this study, not directly taken anywhere. Further explanations were included in the main manuscript under the methods section. The shape files were can be openly accessed by the above-stated link.*

6) In the online submission form, you indicated that [Data is available at the hand of principal investigator and can access if required].
All PLOS journals now require all data underlying the findings described in their manuscript to be freely available to other researchers, either
1. In a public repository
2. Within the manuscript itself
3. Uploaded as supplementary information.
This policy applies to all data except where public deposition would breach compliance with the protocol approved by your research ethics board. If your data cannot be made publicly available for ethical or legal reasons (e.g., public availability would compromise patient privacy), please explain your reasons by return email and your exemption request will be escalated to the editor for approval. Your exemption request will be handled independently and will not hold up the peer review process, but will need to be resolved should your manuscript be accepted for publication. One of the Editorial team will then be in touch if there are any issues.

*Responses: We agree*

7) We note that G Ch was removed from the Author list and  G C F was added. For both these Authors, the email remains  the same [i.e [gemechiscc@gmail.com](mailto:gemechiscc@gmail.com)] but the names are different [G Ch , G C F ]. DG is added to the Authors

*Responses: We added Mr. DG as we missed him because of a type error on our first manuscript and all co-authors agreed to add him. On the other hand, we never removed Authors, but we added grandfather on GC then became GCF and changed the order of the Authors based on their contribution, in case Mr.GCF came to the last order (he had high contributions). We, all Authors have discussed the change we made before we sent the manuscript. We changed the name of our title as the current title is more specific to show what we did and based on the comments given by reviewers (We put it on the document of point-to-point response to reviewers).*

*Responses on the Order of the Authors:* ***Author (PI):*** *Bikila Negesa Gobena^1^;* ***Co-Authors****: Teshome Kabeta^2^, Gemechu Chemeda Feyisa^3^, Birhanu Kenate^4,^ Gemechu Shumi^5^, Fantahun Workine^6^, Haimanot Workineh^7^, Ebise Djirata^8^, and Dabesa Gobena^9^*

C. **Reviewer Comments**

**Reviewer # 1**

- Were correct statistical analysis used to support conclusions?
  No

*Responses: We accepted the comment, and revisions were made: The* conclusion parts are re-written based on the results from the statistical analysis and incorporated into *the revised manuscript.*

- Are there concerns about ethical or regulatory requirements being met?
  No

*Responses: We accepted the comment, and revisions were made to the* Ethical processes and regulatory systems are re-written and incorporated into *the revised manuscript.*

1. The title of the manuscript is general; the authors must specify depending on the innovation of the research.

*Responses: “We accepted the comment, and a modification was made to the* title of the manuscript in *the changed as “****Unraveling the dynamics of Dengue in Metahara town, East Shewa, Oromia, Ethiopia, 2023****“ in the revised manuscript”*

2. The references used in the introduction are insufficient. I suggest adding other works, in which different factors related to the outbreak of dengue cases are mentioned

*Responses: A revision was made to the* reference in *the revised manuscript.*

3. Add in the section Data processing and analysis, the related equations for the calculation of Chi-square tests, and Binary logistic regression.

*Responses: Revision was made to the* data processing, and a clear equation used for chi-square and binary logistic regression was used in *the revised manuscript.*

4. In Figure 2, "Date of onset" is superimposed on the x-axis.

*Responses: We accepted the comment, and revision was made* *the revised manuscript.*

5. Present the multivariate analysis mentioned in the discussion section graphically for the greater interest of readers.

*Responses: Thank you for your comment. We accepted the comment, and the revision resolved* in *the revised manuscript.*

6. -Are the figures (Tables, Images) of sufficient quality for clarity?
   No

*Responses: Thank you for genuine comments, we revised the manuscript as “Tables and image (Map) charged as per the standard( Removing internal cell except the head in table, Image resolution was changed to 300dpi)”*

**Reviewer #2**

- Page 4, paragraph 2: I suggest changing “September2023” to “September 2023.”

*Responses: Thank you for your comment. We accepted the comment, and revision was made in* the *revised manuscript.*

- Page 4, paragraph 3: I suggest changing “The study design was descriptive study” to “Study design: A Descriptive study of a dengue outbreak was conducted followed…”.

*Responses: Thank you for your comment. We accepted the comment, and revision was made in* the *revised manuscript.*

- Page 4, paragraph 4, sample size section: the authors referred to “…odds ratio of 0.31 for no close contact in the previous two weeks…”. Also, they said, “control sample people of Metahara town who were neighbors to a case but did not develop signs and symptoms of DENGUE were included as controls”. In addition, the variable close contact was not shown in the results. I suggest clarifying the definition of close contact and showing the results of the analysis of this variable.

*Responses: Thank you for your comment and here below are the clarification points.*

*“When we calculate the sample size for the current study, we considered the independent variables that was significant and had been widely used, readily available and sufficient statistical power in previous studies. The independent variable” no close contact in the previous two weeks was fulfilled the criteria for independent variable selection we set. Even though it is not a potential factors for the current study, its inclusion in the sample size calculation can provide a more comprehensive understanding of the study’s potential outcomes”*

- Page 5, paragraph 2, sampling procedure section: I suggest specifying the sampling procedure for controls (sampling was in the same block? Or 250 mt around?). In addition, the authors said, “the considering dengue incubation time after dengue cases were discontinued.” I suggest specifying the time.

*Responses: We accepted the comment, and revision was made in* the *revised manuscript.*

Page 5, paragraph 3, The eligibility criteria section: I suggest changing “The eligibility criteria were as follows;” to “Eligibility criteria: (1)…”. In addition, considering that controls were selected in case’s neighbor, I suggest changing “(2) All case/ controls who were not residents of Metahara town” to “(2) All case who were not residents of Metahara town.”

*Responses: Thank you very much! revision was made in* the *revised manuscript as “***The eligibility criteria:** (1) all confirmed and epidemiologically linked Dengue cases from both public and private healthcare facilities starting from August 3 to September 5 /2023, were included as cases, (2) people of Metahara town who were in the same block to a case but did not develop signs and symptoms of Dengue were included as controls. Exclusion criteria: (1) Suspected Dengue patients who were seriously ill, (2) All cases who were not residents of Metahara town.”

- Page 5, paragraph 5, Data collection procedures section: “The data was collected through face to face interview using questionnaire, Health facilities report by using line list, laboratory test result, Environmental investigation, and document review”. This information was repeated in the same paragraph. I suggest eliminating it.

*Responses: We accepted the comment, and a revision was made in* the *revised manuscript.*

Page 6, paragraph 1, Definition terms: I suggest moving this information to “Eligibility criteria” section. In addition, I suggest clarifying the definition of confirmed dengue case because in sample size section the authors said, “it confirmed with Real-Time Polymerase Chain Reverse transcriptase (RT-PCR) or Rapid Diagnostic Test (RDT)…” and in “confirmed cases” did not mention RT-PCR. Also, it is important to clarify de definition of “epidemiologically connected” (time and space definition).

*Responses: Thank you for your comment. We accepted the comment, and revision was made in* the *revised manuscript. However, we put this part here based on the journal guideline.*

- Page 6, paragraph 4: I suggest eliminating the sentences “Finally, the study result was displayed by using graph, figure and table.”

*Responses: Thank you for your comment. We accepted the comment, and revision was made in* the *revised manuscript*

- Figures and tables should be cited in the text.

*Responses: Thank you for your comment. We accepted the comment, and revision was made in* the *revised manuscript*

- Page 7, paragraph 1: I suggest changing “dengue serotype III” to “DENV-3”.

*Responses: Thank you for your comment. We accepted the comment, and revision was made in* the *revised manuscript*

- Page 7, paragraph 1: I suggest avoiding the mention of the name of the index case.

*Responses: Thank you for your comment. We accepted the comment, and revision was made in* the *revised manuscript as “The team investigated the index case retrospectively on July 25/2023, a 12-year-old female patient*”

- Page 7, paragraph 1: The name of the mosquito must be correct throughout the text and written in Italic letters. It is “*Aedes aegpti*”.

*Responses: Thank you for your comment. We accepted the comment, and revision was made in* the *revised manuscript.*

- Page 7, paragraph 2: The year must be correct (2025 to 2023).

*Responses: Thank you for your comment. We accepted the comment, and revision was made in* the *revised manuscript*

- Page 8, paragraph 1: The year must be correct (2024 to 2023)

*Responses: Thank you for your comment. We accepted the comment, and revision was made in* the *revised manuscript*

- Page 8, paragraph 1: The authors mentioned, “On the other hand, the probable date of the outbreak could be between July 27 and July 29, 2023. It is important to note that this was an estimate, as the actual initial exposure and outbreak start date may be earlier or later, depending on factors such as the completeness of case reporting…”, and before they said, “the date of onset was 25th July, 2025 which was index case and in 28th, 2023 was when dengue outbreak was confirmed which the start of the outbreak”. Taking into account this information and the epidemic curve presented (Figure 2), I suggest changing “outbreak start date may be earlier or later” to “outbreak start date may be earlier…” because later is not possible.

*Responses: Thank you for your comment. We accepted the comment, and revision was made in* the *revised manuscript as “On the other hand, the probable date of starting the outbreak might be between July 27 and July 29, 2023. It is important to note that this was an estimate, as the actual initial exposure and outbreak start date may be earlier, depending on factors such as the completeness of case reporting, the sensitivity of the surveillance system, and the potential for undetected or asymptomatic case (figure 2*)”

- Page 8, paragraph 2: According to the Table 1, the attack rate must be correct (718/10,000 for 718/100,000).

*Responses: Thank you for your comment. It revised as “A total of 342 Dengue cases and three deaths with a case fatality rate of 0.88~1% between July 25 to September 5/2023, and the overall attack rate (AR) 7.1/1000. From all cases 6(1.8%) were managed as inpatient whereas 336(98.2%) were treated as outpatients. Male sex was 182(56%), and the age group 25-44 were predominantly 189(55%) affected group respectively (Table 1*).”

- Page 8, paragraph 2: The following sentence is incomplete “From all cases 6(1.8%) were managed as inpatient whereas 336(98.2%).”
  Page 8, paragraph 2: The following sentence is incomplete “From all cases 6(1.8%) were managed as inpatient whereas 336(98.2%).”

*Responses: Thank you for your comment. We accepted the comment, and revision was made in* the *revised manuscript*

- Page 8, paragraph 2: The authors said, “15-44 were predominantly 189(55%) exposed group”. However, the Table 1 shows 25-54. Please clarify it. In addition, I sugest changing “exposed group” to “affected group”.

*Responses: Thank you for your comment. We accepted the comment, and revision was made in* the *revised manuscript*

- Page 9, laboratory investigation section. I suggest eliminating this section because the information was mentioned in “Dengue outbreak verification section”.

*Responses: Thank you for your comment. We accepted the comment, and revision was made in* the *revised manuscript*

- Page 9, paragraph 2, Environmental Investigation section: I suggest moving “ The investigation team noticed breeding site for mosquitoes from house to house, sanitation procedures, the presence of stagnant water, water collection practices, drainage systems, and individual mosquito protection measures, as well as the presence of uncovered water containers, window screening, and empty containers both inside and outside the homes” to methods.

*Responses: Thank you for your comment. We accepted the comment, and revision was made in* the *revised manuscript*

- Table 2. Is it “Not applicable” or “not available”? In addition, I suggest changing “N” to “n”. Also, to simplify, I suggest eliminating “%” of all cells and writing it just once.

*Responses: Thank you for your comment. We accepted the comment, and revision was made to* the *revised manuscript*

- Table 3. Please verify the CI95% in “1.18(0.31-445)”, as it looks too wide.

*Response: We have accepted the comment” There was a point between for and 45 and revision was made in* the *revised manuscript as 0.31-4.45.”*

- Page 12, Coordination section: I suggest including the dates of the activities.

*Responses: Thank you for your comment. We accepted the comment, and revision was made in* the *revised manuscript*

- Page 13, surveillance section: I suggest including dates of the activities.

*Responses: Thank you for your comment. We accepted the comment, and revision was made in* the *revised manuscript*

- Page 13, vector control activities section: I suggest including dates of the activities.

*Responses: Thank you for your comment. We accepted the comment, and revision was made in* the *revised manuscript*

- Page 14, Diagnosis and Case Management: I suggest eliminating “Of 342 lines listed cases 24 were diagnosed for sever dengue and two facility deaths were reported with Dengue shock syndrome”. This information was presented earlier and there is an inconsistency, as the authors previously stated that the deaths were three, but in this part, they mention two.

*Responses: Thank you for your comment. We accept the comment and revision was made.*

- Page 14, Risk Communications and Community Engagement section: I suggest including dates of the activities.

*Response: Thank you for your comment. We accepted the comment, and revision was made in* the *revised manuscript*

- The authors said, “More cases were reported in males than females with increased severity, admission rate and case fatality rate.” However, they only presented the information on attack rate by gender.

*Response: Thank you for your comment and here below are the clarification points.*

“Table 1: revised as sex and age specific attack rate in which the column indicating sex and row indicate age specific attack rate”

- The authors mentioned, “Individuals aged between 15 to 44 years old were predominantly affected”. However, this age range was not presented in the table.

*Response: Thank you for your comment. “Age and sex specific attack rate was calculated in Table 1”*

- In some parts of the text the old classification of dengue (dengue fever, dengue hemorrhagic) is mentioned and in others the current classification (severe dengue). Also, sometimes Dengue is used and other times dengue fever. Please standardize the nomenclature throughout the article.

*Response: Thank you for your comment. We accepted the comment, and revision was made in* the *revised manuscript*

- In the discussion the results are repeated verbatim. I suggest paraphrasing these. Additionally, I suggest including the recommendations section within the discussion. I suggest checking the punctuation and the use of uppercase and lowercase letters.

*Response: Thank you for your comment. We accepted the comment, and revision was made in* the *revised manuscript*

- The manuscript on the study of the first dengue outbreak in Metahara, Ethiopia, and the factors associated with dengue cases is a relevant topic. The author’s present detailed information about the outbreak study and the activities carried out to counteract the impact on the city. However, some aspects of the methodology must be clarified, as well as improving the presentation of the results and avoiding repetition in the text.

*Response: Thank you for your comment. We accepted the comment, and revision was made in* the *revised manuscript*

Reviewer 3

1. Improve the clarity of Figure 1 (Map of Metahara town) by following PLOS-NTD image standards and formats, such as PNG, TIFF, or PDENGUE with a resolution of 300 dpi.

*Response: Thank you for your comment. We accepted the comment, and revision was made in* the *revised manuscript(figure 1)*

2. The description of sampling procedures is lacking details on how blood samples were collected from cases and control groups and transported to the testing laboratory.

*Response: Thank you for your comment. We accepted the comment, and revision was made in* the *revised manuscript as “****Testing and interpretation laboratory****: After notification of the case of dengue virus diseases, eleven samples from 11 patients by using aseptic techniques,4 ml of serum each during the acute period of the disease (0–7 days of symptoms) was collected in serum separated tube and then centrifuged before shipping to avoid hydrolysis using cold pack ensuring that all applicable regulations for sample transport and shipment, stored at -20oc, and sent to the Ethiopian Public Health Institute which is the only national laboratory able to perform RT PCR test for Arboviral disease. Nucleic acid detection assays have three basic steps. These were nucleic acid extraction and purification, amplification of the nucleic acid, and detection and characterization of the amplified product. Four of the 11 samples resulted in positive for DENV-3 and were considered index cases. The STANDARD Q Dengue NS1 Ag test and the procedure for the RDT test were initially we collect 100 ml of whole blood using a micropipette from the suspected cases of patients capillary, we add the collected sample to the sample well of the test device, and after 20 minutes of waiting time, we read the result and interpretation as positive for IgM antibody when control and NS1 line was marked red, and negative when only control line appears red. Otherwise, when the red line did not appear in the control and test line or if it did not appear in the red line of the test line and no red line in the test line it was an invalid result*.”

3. For case definitions: Epidemiological case: Why were positive malaria test cases excluded? The presence of malaria does not rule out concurrent dengue infection, particularly during outbreaks or in endemic tropical settings where malaria-dengue co-infection is common. For example, in Sudan, malaria-dengue co-infections have been recorded up to >10% in some areas (Ahmed et al., 2021) Source.

*Response: Thank you for your comment and here below are the clarification points.*

“We tested antibodies against dengue by using RDT. However, this procedure was done only for patients positive for malaria and showed signs and symptoms resembling dengue cases, because there was a shortage of RDT in the country.”

4. Confirmed case: The definition needs to be revised to follow the WHO dengue guidelines for diagnosis, treatment, prevention, and control (WHO, 2009). IgM antibodies can persist in the blood for up to three months, making it difficult to differentiate between active infection and recent exposures. According to WHO, a confirmed case is RT-PCR+ within 2-7 days after the onset of fever or IgM+ seroconversion in paired sera (acute sample tested against convalescent sample collected after 7 days). The use of RT-PCR in the study should be clearly included in the confirmed case
   definitions. IgM+ alone does not meet the criteria for a confirmed dengue case as per WHO guidelines.

*Response: Thank you for your comment. We accepted the comment, and revision was made in* the *revised manuscript*

5. The description of laboratory testing of samples is missing. Add an explanation of laboratory testing of samples using RDT and later using the RT-PCR method.

*Response: Thank you for your comment. We accepted the comment, and revision was made in* the *revised manuscript as”* ***Epidemiological case****: - Any person lives in Metahara town between July 25 to September 5/2023 presented with a fever lasting 2–7 days with at least one of the following symptoms: a headache, arthralgia, myalgia, rash, or bleeding from any region of the body, along with a negative malaria or presence of IgM antibody positive test were considered as epidemiologically linked cases.*

***Confirmed cases****: A case was said to be confirmed, after RT-PCR laboratory test resulted positive for DENV in laboratory. Control was any person who lives in same block to the patient selected as having Dengue case, but those without signs or symptoms of the disease starting from July 25 to September 5/2023*.”

6. The conclusions are supported by the data presented; however, there is significant redundant information, particularly repeated results and discussion points. For instance, results such as “more cases were reported in males than females with increased severity, admission rate, and case fatality rate” and “the presence of water containers in or around households and not using LLINs during the day were significant risk factors” are repeated without adequate implications being provided. These statements are part of the results rather than the conclusions.

*Response: Thank you for your comment. We accepted the comment, and revision was made in* the *revised manuscript*

7. The author should synthesize these results to provide detailed interpretations and implications. Specifically, the author should explain how these findings can inform public health interventions in Metahara town, particularly in terms of implementing or strengthening vector control, dengue surveillance, and preparedness against future outbreaks, as mentioned in the introduction section.

*Response: Thank you for your comment. We accepted the comment, and revision was made in* the *revised manuscript*

8. The recommendation section should be removed to adhere to the standard "IMRaDC" format; Introduction (the study's purpose), Methods (how the study was conducted and the justification for the chosen methods), Results (what was found), Discussion (the meaning of the findings), and Conclusions (implications and lessons learned from the findings).

*Response: Thank you for your comment. We accepted the comment, and revision was made in* the *revised manuscript*

9. The limitations of the analysis have not been discussed. Additionally, the authors have not sufficiently explained how the data advances understanding of the topic. More synthesis and interpretation are required to address this

*Response: Thank you for your comment. We accepted the comment, and revision was made in* the *revised manuscript*

10. The results are not clearly presented.

*Response: Thank you for your comment. We accepted the comment, and revision was made in* the *revised manuscript “****Limitation*** *In the area, presence of multiple outbreaks like cholera and malaria and the lack of trained health workers about Arbo-virus diseases in the Oromia region and lack of governmental contingency budget for arboviral-related diseases and no partners who had planned activities and budgets for the prevention and control of Arboviral disease at all levels. Low engagement of health workers and inadequate stockpiling of essential supplies, equipment, and medications for case management made the duration of the Dengue outbreak elongate. Integrate outbreak response, prepare Emergency response plan and share it with responsible bodies, and experience sharing and Daily Feedback were used as a backup system in response to these challenges*.”

11. The tables should be formatted according to standard publication rules, e.g., tables should have no internal lines except for headers.

*Response: Thank you for your comment. We accepted the comment, and revision was made in* the *revised manuscript*

12. I recommend recategorizing the age groups to indicate extreme age groups, e.g., 0-4 yrs, 5-14 yrs, 15-24, 25-34, 35-44, 45-54, 55-64, 65+. This is important in the context of dengue immune response and severe disease development, which differ between these age groups, with higher risk observed in children and adults over 60. Immunity for preschool and school children may differ due to varying levels of exposure to dengue infection, with higher risk in the older group (>50) due to enhanced immunity following secondary infections with different dengue types that lead to cross-reactive immune complexes and release of inflammatory agents. These age groups should be consistent in Table 1 and Table 2.

*Response: Thank you for your comment and here below are the clarification points.*

“We discussed the point, but Metahara town is nearest to the dengue-endemic region of the Afar Regional State of Ethiopia. People of the area live partly in the Afar Region and Oromia Regional state. Although we could not identify whether there was a secondary infection, the current outbreak had increased severity and death nearly similar to a secondary infection. So, we agreed to use this type of age category”

13. Section 3.2.3, "Factors associated with dengue outbreak," should be revised to "Risk factors associated with dengue outbreak."

*Response: Thank you for your comment. We accepted the comment, and revision was made in* the *revised manuscript*

14. All figures lack captions (subtitles) and explanations. They should be well-labeled, e.g., Figure A, B, C, etc., and include the source and date, e.g., Source: John et al., 2014.

*Response: Thank you for your comment. We accepted the comment, and revision was made in* the *revised manuscript*

15. There are many grammatical errors and typos in this section that need to be revised.

*Response: Thank you for your comment. We accepted the comment, and revision was made in* the *revised manuscript*

16. All scientific names should be correctly written in italics, e.g., Aedes aegpti.

*Response: Thank you for your comment. We accepted the comment, and revision was made in* the *revised manuscript*
