# Supplementary material for: Unraveling the dynamics of dengue in Metahara town, East Shewa, Oromia, Ethiopia, 2023
Source: PLoS Negl Trop Dis. 2025 Mar 17;19(3):e0012908. doi: 10.1371/journal.pntd.0012908 (PMC11957386; doi:10.1371/journal.pntd.0012908)
Supplement: Attachment — Submitted filename: Response_to_Reviewers.docx [file pntd.0012908.s005.docx]

**Point-by-point responses to reviewer’s and editor's comments**

**Journal**: PLOS Neglected Tropical Diseases

(PNTD-D-24-00552) - [EMID: be8108a824f88701]

**Dear Editors**

We would like to extend our sincere appreciation to you and the reviewers for your thorough evaluations and constructive feedback on our manuscript titled "**Unraveling the Dynamics of Dengue in Metahara Town, East Shewa, Oromia, Ethiopia, 2023**." We have carefully addressed all comments and provided a detailed response to each point raised by the esteemed reviewers. The revisions made to the manuscript are clearly marked in both the tracked changes and clean versions, with modifications highlighted in red for clarity. We look forward to your favorable assessment and consideration of our revised manuscript for publication.

Sincerely,

Bikila Negesa Gobena, Principal Investigator

**Notes from the Additional Editor**

The overall study is very promising. The manuscript is significantly below professional standard. The English needs an expert to review and a scientific editor. The reviewers have also raised this concern and we cannot act as unpaid editor for the authors' shortcomings.

As described by the reviewers, there are errors throughout the manuscript for example,

"Of 50, 16 (32%) cases had heard of Dengue, while 104 (69.3%) of the total controls had heard of ..."
This is not grammatical English. It is one example amongst many.
The nomenclature is erratic across the manuscript,
For example, DNV should be DENV
RT PCR should be RT-PCR
Dengue elsewhere DENGUE. There reality is the correct spelling is dengue.
The manuscript is strewn with errors.

The additional issues are quality of the charts is very basic Excel, the GIS is not sufficient quality. The bar chart is not a good representation of the data. The tables should be converted to charts and the tables transferred to the appendix. The core analytics should be presented as a table, as the authors have done.
I am also concerned about the failure to reference many notable studies in dengue epidemiology in Africa and especially South East Asia.
I cannot let the manuscript past the editorial process until it is of sufficient standard and instruct the authors' to seek external support including expert support and scientific editing to clear up the manuscript. There is no question this an emergent infection to Ethiopia, the authors are experts, have performed a competent and this of significant public health interest.

**Authors’ Responses:**

We appreciate your constructive feedback regarding the need of our manuscript revision for: grammatical error, the nomenclature is erratic and the low quality of: chart, GIS map and table below the PLOS journal standard in the manuscript.

In response, we have conducted a thorough review of the entire document and have made the necessary revisions to address all grammatical errors identified. We have ensured that the language is clear and precise, enhancing the overall readability of the manuscript. We believe these edits contribute to the clarity and professionalism of our work.

We have carefully reviewed the entire document and made the following corrections to the nomenclature errors in the manuscript: Changed all instances of "DNV" to "DENV", Corrected "RT PCR" to "RT-PCR" and standardized the usage of "Dengue" to "dengue" throughout the manuscript. These revisions have been implemented to ensure consistency and adherence to scientific nomenclature standards. We believe these changes enhance the clarity and professionalism of our manuscript.

In response, we have made several revisions to enhance clarity and adherence to publication standards: We have revised the histogram to improve its presentation, ensuring it effectively conveys the data trends. The map has been updated to meet PACE standards, enhancing its clarity and usability for readers. We have reformatted the tables to align with the PLOS journal standards, ensuring consistency with the journal's requirements. While we considered converting some tables into charts for better visualization, we determined that this change was not feasible due to the complexity of the data. Therefore, we have retained the original format where appropriate. The table that was previously sent as figure, it has been added in an appendix for better organization and accessibility.

**Reviewers' Comments:**

Dear Reviewers,

We would like to express our sincere gratitude for the time and effort you have dedicated to reviewing our manuscript. Your insightful comments and constructive feedback have been invaluable in enhancing the quality and clarity of our work. We appreciate your thorough evaluation of our study, and we have carefully considered each of your suggestions.

In response to your comments, we have made several revisions to the manuscript, including clarifications in the methodology, additional analyses, and improvements in the presentation of our findings. We believe these changes address your concerns and strengthen the overall contributions of our research.

We are committed to ensuring that our work meets the highest standards of scientific rigor, and we hope that the revisions we have made reflect this commitment. Thank you once again for your thoughtful feedback. We look forward to your further evaluation of our revised manuscript.

Sincerely,

Bikila Negesa Gobena, Principal Investigator

 Reviewer's Responses to Questions

**Key Review Criteria Required for Acceptance?**
As you describe the new analyses required for acceptance, please consider the following:
**Methods**
-Are the objectives of the study clearly articulated with a clear testable hypothesis stated?
-Is the study design appropriate to address the stated objectives?
-Is the population clearly described and appropriate for the hypothesis being tested?
-Is the sample size sufficient to ensure adequate power to address the hypothesis being tested?
-Were correct statistical analysis used to support conclusions?
-Are there concerns about ethical or regulatory requirements being met?
 Reviewer #1: (No Response)

Reviewer #2: In general, I agree. However, please see my comments below.

**Results**
-Does the analysis presented match the analysis plan?
-Are the results clearly and completely presented?
-Are the figures (Tables, Images) of sufficient quality for clarity?
 Reviewer #1: (No Response)

Reviewer #2: In general, I agree. However, please see my comments below.

**Conclusions**
-Are the conclusions supported by the data presented?
-Are the limitations of analysis clearly described?
-Do the authors discuss how these data can be helpful to advance our understanding of the topic under study?
-Is public health relevance addressed?
 Reviewer #1: (No Response)

Reviewer #2: The limitations mentioned by the authors are quite general. They should be specific to the study.

***Response:*** *Thank you for your valuable feedback on our manuscript. We appreciate the reviewers’ insights regarding the limitations of our study. In response, we have revised the section discussing the limitations to provide a clearer and more specific context related to our findings in the manuscript*.

**Editorial and Data Presentation Modifications?**
Use this section for editorial suggestions as well as relatively minor modifications of existing data that would enhance clarity. If the only modifications needed are minor and/or editorial, you may wish to recommend “Minor Revision” or “Accept”.

Reviewer #1: (No Response)

**Reviewer #2**: 1. It is important to conduct a thorough proofreading of the article, as there are several issues throughout the text, including misplaced full stops in sentences, inappropriate use of capital letters, punctuation errors before and after references, and instances where references are written without spaces between the preceding word and the parentheses.

**Response**: *We sincerely appreciate the reviewers' attention to detail in their evaluation of our manuscript. In response to the feedback regarding proofreading and formatting issues, we have undertaken a thorough review of the entire document. We meticulously proofread the manuscript to identify and correct all misplaced full stops and punctuation errors. We reviewed instances of inappropriate use of capital letters and made necessary adjustments to ensure consistency and adherence to grammatical standards. We specifically addressed punctuation errors before and after references, ensuring that all citations conform to the required style guidelines.*

2. The following text is repeated in “Study Area and Period” section. Please remove the duplicate content. “Based on the town administrative report of 2022/23, the town population was 47,661, one health center, two health posts, and 13 private clinics. The country has made significant strides in improving its disease surveillance system. Although Arboviral disease outbreaks like Yellow fever, Dengue, Chikungunya, and Sand Fly Fever in Ethiopian neighboring countries like Sudan, Eritrea, Kenya, and Djibouti, there are still limitations in enhancing arboviral disease surveillance in Ethiopia”.

***Response:*** *We would like to extend our gratitude to the reviewers for their insightful comments regarding the presence of duplicated content in our manuscript.*

*In response to this feedback, we have carefully reviewed the entire manuscript and have removed all instances of duplicated content.*
3. Please correct “anti-DENVI gG/IgM” to “anti-DENV IgG/IgM”.

***Response:*** *We appreciate the reviewers' comments. In response, we have corrected it in the revised manuscript.*

4. Please correct in Results, 1 paragraphac “DNV-3” to “DENV-3”.

***Response:*** *We appreciate the reviewers' comments. In response, we have corrected it in the revised manuscript.*

5. Please correct “DENGUE” to “Dengue”.

***Response:*** *We appreciate the reviewers' comments. In response, we have corrected it through all contents of the revised manuscript.*

6. Please correct “Arbo-virus diseases” to “arboviral diseases”

***Response:*** *We appreciate the reviewers' comments. In response, we have corrected it in the revised manuscript.*

**Summary and General Comments**
Use this section to provide overall comments, discuss strengths/weaknesses of the study, novelty, significance, general execution and scholarship. You may also include additional comments for the author, including concerns about dual publication, research ethics, or publication ethics. If requesting major revision, please articulate the new experiments that are needed.

Reviewer #1: (No Response)

**Reviewer #2**: The authors addressed most off the comments. However, there are still some that were not taken into account, and I believe they are important.
1. In the sample size section, the authors referred to an “odds ratio of 0.32 for no close contact in the previous two weeks”. They also clarified that the control group consisted of “any person who lives in same block to the patient selected as having Dengue case, but those without signs or symptoms of the disease starting from July 25 to September 5/2023”. Given this, it appears that all controls were either in relatively close contact or entirely unexposed (i.e., no close contact because they do not live in the same household as the cases). As a result, this variable becomes a constant among the controls, making impossible to calculate an OR, as there would be zero exposed control. In addition, the variable “close contact”, which was the primary exposure variable used to calculate the sample size, does not appear in the results. I recommend either clarifying the definition of “close contact” and presenting the analysis of this variable in the results or modifying the text to reflect more general information for any variable with a similar frequency distribution.

**Response**: *We acknowledge the concerns you raised about the calculation of the odds ratio (OR) in relation to our control group. To address your comments, we have added a clear definition of “close contact” in the methods section. Specifically, we define* ***Close contact****: is defined as being within a distance of 1 meter from a confirmed dengue case for at least 15 minutes within the last two weeks July 25 to September 5, 2023.* ***No close contact****: indicates that an individual has not been within this proximity of a confirmed case during the same timeframe July 25 to September 5, 2023. This clarification aims to provide a better understanding of how we categorized participants in our study. We have now included an analysis of the “close contact” variable in the results section. We conducted a detailed examination of how this variable influenced dengue transmission among cases and controls. The analysis includes descriptive statistics which were previously omitted.*
2. The name of the mosquito should be consistent and correctly formatted throughout the text in Italic letters: Aedes aegypti. For example, in the first paragraph of the results, “Aedes Aegpti”, is misspelled, and on page 14, “anopheles Aegyptus” is incorrectly used. Please ensure that the correct species name, Aedes aegypti, is used consistently (<https://wrbu.si.edu/vectorspecies/mosquitoes/aegypti> ; <https://www.ncbi.nlm.nih.gov/Taxonomy/Browser/wwwtax.cgi?mode=info&id=7159>

***Response:*** *We appreciate the reviewers' comments. In response, we have corrected it in the revised manuscript.*
3. On page 12, paragraph 1, the year should be corrected from 2024 to 2023."

***Response:*** *We appreciate the reviewers' comments. In response, we have corrected it in the revised manuscript.*
4. On page 12, paragraph 2, the authors state “15-44 were predominantly 189(55%) exposed group”, but Table 1 shows the age range as 25-54. Please correct this discrepancy. The same error appears in the Discussion section on page 17.

*Response: We sincerely apologize for the typographical error you identified in our manuscript. We have made the necessary corrections in the revised version to ensure clarity and accuracy.*
5. Table 2. Is it “Not applicable” or “not available”?

Response: Dear Reviewer, we appreciate your insights, but we apologize for any confusion caused by the phrasing of "Not applicable" versus "Not available."

To address your concern, we have made the following revisions: We have included an additional variable in Table 2 as suggested. Furthermore, we have reformatted the table for improved readability and clarity, ensuring that all relevant information is presented clearly. We believe these changes will enhance the overall quality of our manuscript and address your concerns effectively. Thank you once again for your constructive feedback.

Additional comments:
1. In “Risk factors associated with Dengue outbreak” section (page 14). I suggest changenig “Dengue patients who did not use Long-Lasting Insecticide Nets (LLINs) (LLINs) during daytime were 9.07 times more likely risk of developing Dengue (AOR= 9.07, 95% CI: 2.05-40.06) compared to those who did not use it.” to “Individuals who did not use Long-Lasting Insecticide Nets (LLINs) (LLINs) during daytime were 9.07 times more likely risk of developing Dengue (AOR= 9.07, 95% CI: 2.05-40.06) compared to those who use it”.

***Response:*** *We appreciate the reviewers' comments. In response, we have corrected it in the revised manuscript.*
2. In Table 2, the suggestion was to remove the “%” symbol, not the relative frequencies. Please retain the relative frequencies, for example, 25 (50) and 41 (41).

***Response:*** *We appreciate the reviewers' comments. In response, we have corrected it in the revised manuscript.*
3. I suggest moving the limitations section to the Discussion section or placing before the Conclusion section.

***Response:*** *We appreciate the reviewers' comments. In response, we have corrected it in the revised manuscript.*

PLOS authors have the option to publish the peer review history of their article ([what does this mean?](https://track.editorialmanager.com/CL0/https:%2F%2Fjournals.plos.org%2Fplosntds%2Fs%2Feditorial-and-peer-review-process%23loc-peer-review-history/1/010f0192c2391639-1e546806-4336-4b8f-964b-5c639b21d155-000000/1rxQcVt1gcT7IdkLJgKnMWMF1724OMzv6pRA30yllO0=182)). If published, this will include your full peer review and any attached files.


If you choose “no”, your identity will remain anonymous but your review may still be made public.


**Do you want your identity to be public for this peer review?** For information about this choice, including consent withdrawal, please see our [Privacy Policy](https://track.editorialmanager.com/CL0/https:%2F%2Fwww.plos.org%2Fprivacy-policy/1/010f0192c2391639-1e546806-4336-4b8f-964b-5c639b21d155-000000/jVpo9qfk9IFMhNgbnWFVIgYUIY3hg9sGKYFvrnbtaSg=182).

Reviewer #1: No

Reviewer #2: **Yes:** Ruth Aralí Martinez Vega

[NOTE: If reviewer comments were submitted as an attachment file, they will be attached to this email and accessible via the submission site. Please log into your account, locate the manuscript record, and check for the action link "View Attachments". If this link does not appear, there are no attachment files.]

**Figure resubmission:**

 While revising your submission, please upload your figure files to the Preflight Analysis and Conversion Engine (PACE) digital diagnostic tool, <https://pacev2.apexcovantage.com/>. PACE helps ensure that figures meet PLOS requirements. To use PACE, you must first register as a user. Registration is free. Then, login and navigate to the UPLOAD tab, where you will find detailed instructions on how to use the tool. If you encounter any issues or have any questions when using PACE, please email PLOS at [figures@plos.org](mailto:figures@plos.org). Please note that Supporting Information files do not need this step. If there are other versions of figure files still present in your submission file inventory at resubmission, please replace them with the PACE-processed versions.

**Reproducibility:**

To enhance the reproducibility of your results, we recommend that authors of applicable studies deposit laboratory protocols in [protocols.io](http://protocols.io/), where a protocol can be assigned its own identifier (DOI) such that it can be cited independently in the future. Additionally, PLOS ONE offers an option to publish peer-reviewed clinical study protocols. Read more information on sharing protocols at <https://plos.org/protocols?utm_medium=editorial-email&utm_source=authorletters&utm_campaign=protocols>
