# Supplementary material for: Unraveling the dynamics of dengue in Metahara town, East Shewa, Oromia, Ethiopia, 2023
Source: PLoS Negl Trop Dis. 2025 Mar 17;19(3):e0012908. doi: 10.1371/journal.pntd.0012908 (PMC11957386; doi:10.1371/journal.pntd.0012908)
Supplement: Attachment — Submitted filename: Response to Reviewers.docx [file pntd.0012908.s006.docx]

**Point-by-point responses to reviewer’s and editor's comments**

**Journal**: PLOS Neglected Tropical Diseases

(PNTD-D-24-00552R2) - [EMID: be8108a824f88701]

**Dear Editors**

We would like to extend our sincere appreciation to you and the reviewers for your thorough evaluations and constructive feedback on our manuscript titled "**Unraveling the Dynamics of Dengue in Metahara Town, East Shewa, Oromia, Ethiopia, 2023**." We have carefully addressed all comments and provided a detailed response to each point raised by the esteemed reviewers. The revisions made to the manuscript are clearly marked in both the tracked changes and clean versions, with modifications highlighted in red for clarity. We look forward to your favorable assessment and consideration of our revised manuscript for publication.

Sincerely,

Bikila Negesa Gobena, Principal Investigator

**Additional Editor Comments:**

I requested a full revision of the English in the manuscript. The authors have not performed this to anything close to a satisfactory standard.
The authors are therefore requested to go through each of the 43 comments concerning grammatical and syntax errors, and issues with clarity for the Abstract, Introduction, Methods, Results and Discussion and address each in turn. On the revision state which changes have been made and give clear justification if not performed. The revision will be checked in detail to ensure all changes have been incorporated.

We appreciate your constructive comments regarding the need of our manuscript revision. We have conducted a thorough review of the entire document and have made the necessary revisions to address all the errors identified. We believe these edits contribute to the clarity and professionalism of our work.

Abstract:
* "Dengue, a mosquito-borne viral disease, has increasingly been reported in Ethiopia, with a suspected outbreak occurring in Metahara town, Oromia Regional State, in July 2023."
Issue: The sentence is clear but could benefit from greater specificity about "increasingly been reported."
Suggestion: Specify the timeframe or trends (e.g., "since [year], dengue cases have shown a marked increase in Ethiopia").

***Responses***: *Thank you for your genuine suggestion. We included it in the revised manuscript as”* Since 2013, dengue cases have shown a marked increase in Ethiopia”

* "This study aimed to confirm the outbreak, identify associated risk factors, describe epidemiological characteristics, and implement control measures."
Suggestion: Simplify by avoiding repetition: "This study aimed to confirm and characterize the outbreak, identify risk factors, and implement control measures."

***Responses****: Thank you once again! We arranged as*” This study aimed to confirm and characterize the outbreak, identify risk factors, and implement control measures” *in the revised manuscript.*

* "A descriptive study followed by an unmatched case-control design (1:2 case-to-control ratios) was conducted."
Issue: "Followed by" is slightly unclear. Were the descriptive study and case-control study distinct phases or conducted concurrently?
Suggestion: Rephrase for clarity, e.g., "A descriptive study and an unmatched case-control study (1:2 case-to-control ratio) were conducted."

***Responses****: We accept and revised as”* The investigators conducted a descriptive study with an unmatched case-control design, using a one-to-two ratio of cases to controls” *in the manuscript*

* "Data were collected using Kobo Collect and analyzed with SPSS version 26 for statistical analysis and Microsoft Excel 2013 for descriptive statistics."
Issue: Repetition of "for statistical analysis."
Suggestion: Simplify: "Data were collected using Kobo Collect, analyzed with SPSS version 26, and summarized with Microsoft Excel 2013."

***Responses****: Thank you very much, we revised as”* The investigators conducted a descriptive study with an unmatched case-control design, using a one-to-two ratio of cases to controls” *in the manuscript.*

"The strength of associations was measured using crude odds ratios (OR) and adjusted odds ratios (AOR), with statistical significance defined at p-values ≤ 0.05."
* Issue: Clarity is good, but the text could be condensed.
Suggestion: "Crude and adjusted odds ratios (OR and AOR) were used to measure associations, with p-values ≤ 0.05 indicating significance."

***Responses****: Thank you very much, we revised as”* Crude and adjusted odds ratios (OR and AOR) were used to measure associations, with p-values ≤ 0.05 indicating significance” *in the manuscript.*

* "The investigation confirmed 342 cases of dengue, resulting in an attack rate of 7.1 per 1,000 populations, with a case fatality rate of 0.88%."
Issue: "Populations" should be singular ("population").
Suggestion: "The investigation confirmed 342 dengue cases, corresponding to an attack rate of 7.1 per 1,000 population and a case fatality rate of 0.88%."

***Responses****: We appreciate your constructive suggestion and we revised it as”* The investigation confirmed 342 dengue cases, corresponding to an attack rate of 7.1 per 1,000 population and a case fatality rate of 0.88%” *in our manuscript.*

* "Factors associated with the dengue were: not using long-lasting insecticide nets during the daytime was a ninefold increased likelihood of dengue outbreak..."
Issue: Awkward phrasing and incorrect structure.
Suggestion: "Significant risk factors included not using long-lasting insecticide nets during the daytime (9-fold increased likelihood) and having open water containers (5-fold increased likelihood)."

***Responses****: We appreciate your constructive suggestion and we revised it as”* Significant risk factors included not using long-lasting insecticide nets during the daytime (9-fold increased likelihood) and having open water containers (5-fold increased likelihood” *in our manuscript.*

* "The dengue outbreak in Metahara town is significantly influenced by epidemiological, entomological, and environmental factors, with Aedes aegypti identified as the primary vector."
Suggestion: Streamline for clarity: "The dengue outbreak in Metahara town was driven by epidemiological, entomological, and environmental factors, with Aedes aegypti as the primary vector."

***Responses****: We appreciate your constructive suggestion and we made revision as”* The dengue outbreak in Metahara town was driven by epidemiological, entomological, and environmental factors, with Aedes aegypti as the primary vector” *in our revised manuscript.*

"Hence, strategies combining vector control measures with community education, promoting long-sleeved clothing, raising awareness about dengue transmission, and establishing robust surveillance systems were recommended."
Suggestion: Avoid redundancy: "Strategies including vector control, community education, promotion of protective clothing, and improved surveillance were recommended."

***Responses****: We appreciate your constructive suggestion and we made revision as”* Strategies including vector control, community education, promotion of protective clothing, and improved surveillance were recommended” *in our revised manuscript*

* "The Authors conducted a detailed and comprehensive investigation..."
Issue: "Authors" should not be capitalized. The phrase "detailed and comprehensive" is redundant.
Suggestion: "The authors conducted an investigation..."

***Responses****: Thank you, we made revision to your suggestion as* “The authors conducted an investigation into how the disease spread, what factors enhanced the outbreak, and the strategy to manage the dengue outbreak” *in our revised manuscript*

* "Intermittent rains, inappropriate water storage practices, and numerous old tires in town before the outbreak had created ideal breeding grounds for these mosquitoes."
Issue: Verb tense inconsistency ("had created").
Suggestion: Use the simple past tense: "...created ideal breeding grounds for mosquitoes."

***Responses****: Thank you, we made revision to your suggestion as* “Intermittent rains, inappropriate water storage practices, and numerous old tires in town before the outbreak created ideal breeding grounds for these mosquitoes” *in our revised manuscript*

* "The outbreak spread rapidly, with hundreds of cases reported in a short period."
Suggestion: Specify the timeline if possible: "...in a span of [timeframe]."
* "A multi-faceted approach that was enhancing disease surveillance..."
Issue: Incorrect verb form.

***Responses****: Thank you very much for you constructive suggestion, we accepted your comment and made revision as* “The outbreak escalated quickly, with hundreds of cases reported between July 25 and September 5, 2023” *in our revised manuscript*

Suggestion: "A multi-faceted approach that enhanced disease surveillance..."
* "The author's comprehensive investigation provides valuable insights..."

***Responses****: Thank you, we revised as* “The author's comprehensive approach, such as active surveillance and case reporting, vector control activities, improved access to diagnostic testing and clinical management and launching public awareness campaigns, provided valuable insights into outbreak control” *in the manuscript*

Issue: "Author's" is incorrect; use "authors'."
Suggestion: "The authors' investigation provides..."

***Responses****: Thank you, we revised as* “The authors’ investigation provides the challenges faced by the health system in managing the outbreak, such as limited resources, infrastructure constraints, and coordination issues between different agencies” *in the manuscript*

Introduction, Methods and Results
* The statement "Dengue can cause asymptomatic infections as well as life-threatening complications such as dengue hemorrhagic fever and shock syndrome" is redundant as the previous sentence already conveys the same idea.

***Response:*** *Thank you for your valuable feedback on our manuscript. We appreciate the* Additional Editor Comments*’ insights regarding the limitations of our study.*

*In response, we have removed the redundant part and combine together in the manuscript*.

Suggested revision: Combine the sentences for clarity and conciseness.
* "Since 2013, the dengue outbreak has increased in the healthcare system of Ethiopia" could be rephrased for accuracy and clarity as "Since 2013, dengue outbreaks have increasingly burdened Ethiopia's healthcare system."

***Response:*** *Thank you for your valuable feedback on our manuscript. We appreciate the* Additional Editor Comments*’ insights regarding the limitations of our study.*

*In response, we have revised as”* Since 2013, dengue outbreaks have increasingly burdened Ethiopia's healthcare system” *in the manuscript*

* "The first confirmed case was identified in Dire Dawa City, eastern Ethiopia ten years ago" requires a comma after "Ethiopia."

***Response:*** *Thank you for your valuable suggestion. We have revised as”* The first confirmed case was identified in Dire Dawa City, eastern Ethiopia, ten years ago” *in the manuscript*

* Phrases such as "presence of anti-DENV IgG/IgM antibodies in patients who are feverish" could be simplified to "detection of anti-DENV IgG/IgM antibodies in febrile patients" for improved readability.

***Response:*** *Thank you for your valuable suggestion. We have revised as”* The detection of anti-DENV IgG/IgM antibodies in feverish patients helped identify circulating dengue infections across the country” *in the manuscript*

* The section discussing risk factors and LLIN utilization transitions abruptly. Consider reorganizing the paragraph to discuss vector control measures more cohesively.

***Response:*** *Thank you for your valuable suggestion. We have* Edited the previous and exclusively we added new paragraph” *in the manuscript*

* Some statements, like "Ethiopia has made significant progress in enhancing its disease surveillance system," lack citations for substantiation.

***Response:*** *Thank you for your valuable suggestion.”* we add reference” *in the manuscript*

* The vaccine paragraph lacks clarity. Revise as: "While dengue is vaccine-preventable, vaccines like Dengvaxia and Qdenga are not yet part of Ethiopia's immunization program. The recent WHO prequalification of Qdenga may facilitate future availability."

***Response:*** *Thank you for your valuable suggestion. We have revised as”* While dengue is vaccine-preventable, vaccines like *Dengvaxia* and *Qdenga* are not yet part of Ethiopia's immunization program. The recent WHO prequalification of *Qdenga* may facilitate future availability” *in the manuscript*

* The description of Metahara Town's climate and geography could be streamlined for focus.

Suggested revision: "Metahara Town, in the East Shewa Zone of Oromia, Ethiopia, lies 193 km from Addis Ababa and experiences a hot, partly cloudy climate with annual temperatures ranging from 67°F to 93°F."

***Response:*** *Thank you for your valuable suggestion. We have revised as”* Metahara Town, in the East Shewa Zone of Oromia, Ethiopia, lies 193 km from Addis Ababa and experiences a hot, partly cloudy climate with annual temperatures ranging from 67°F to 93°F” *in the manuscript*

* "Using QGIS software, we created a map of the research area by evaluating and overlaying various data layers" is awkward. Simplify to: "We used QGIS software to create a research area map by overlaying data layers."

***Response:*** *Thank you for your valuable suggestion. We have revised as”* We used QGIS software to create a research area map by overlaying data layer” *in the manuscript*

* Avoid passive phrases like "The sampling frame was created." Instead: "We created the sampling frame."

***Response:*** *Thank you for your valuable suggestion. We have revised as”* We developed the sampling frame based on a line list of dengue patients” *in the manuscript*

* "Control was any person who lives in the same block with the case" should be "A control was defined as any person living in the same block as a case."

***Response:*** *Thank you for your valuable suggestion. We have revised as”* A control was defined as any person living in the same block as a case, but no signs or symptoms of the disease during the study period” *in the manuscript*

* Specify the validity of the STANDARD Q Dengue NS1 Ag test for local conditions to enhance credibility.

***Response:*** *Thank you for your valuable suggestion. We have revised as”* The STANDARD Q Dengue NS1 Ag test demonstrates strong validity, with a sensitivity of 92.9% and specificity of 98.7%, effectively detecting dengue virus infections in serum, plasma, or whole blood samples” *in the manuscript*

* Replace "4 ml of serum during the acute phase of the disease (0–7 day’s post-symptom onset)" with "4 mL of serum during the acute phase (0–7 days post-symptom onset)."

***Response:*** *Thank you for your valuable suggestion. We revised in the manuscript*

* Combine eligibility and exclusion criteria for better organization.

***Response:*** *Thank you for your valuable comment. We revised in the manuscript*

* "All cases who were not residents of Metahara town" should be "All non-resident cases of Metahara Town."

***Response:*** *Thank you for your valuable comment. We revised in the manuscript*

* The phrase "mandated for the outbreak" is unclear. Replace with "required for outbreak investigation."

***Response:*** *Thank you for your valuable comment. We revised in the manuscript*

* State explicitly whether verbal consent was sufficient under the ethical clearance.

***Response:*** *Thank you for your valuable suggestion. We have revised as”* During the investigation of the dengue outbreak, we chose to obtain verbal consent as a means to quickly engage participants in light of the pressing public health crisis. This method allowed us to inform individuals about the study while still adhering to ethical guidelines that permit verbal consent when written consent is not feasible. Additionally, we made sure to document the verbal consent to uphold transparency and accountability throughout the entire process” *in the manuscript*

* "Primary data collection was performed using face-to-face interviews via the Kobo Collect mobile application" could be simplified as: "We collected primary data using face-to-face interviews through the Kobo Collect mobile app."

***Response:*** *Thank you for your valuable suggestion. We have revised as”* We collected primary data using face-to-face interviews through the Kobo Collect mobile app” *in the manuscript*

* The description of the questionnaire's components is lengthy. Use bullet points for clarity:

***Response:*** *Thank you for your valuable comment. We revised in the manuscript*

* Use consistent phrasing for definitions, e.g., "An epidemiological case is defined as..."

***Response:*** *Thank you for your valuable comment. We revised in the manuscript*

* In "Close contact is defined as being within a distance of 1 meter," change "is defined" to "was defined" to maintain past tense consistency.

***Response:*** *Thank you for your valuable comment. We revised in the manuscript*

* Replace "Data was checked for completeness, coded, cleaned" with "Data were checked for completeness, coded, and cleaned."

***Response:*** *Thank you for your valuable comment. We revised in the manuscript*

* The statistical methods need simplification for broader readability. For instance, "We used binary logistic regression to analyze the data and determine associations."

***Response:*** *Thank you for your valuable comment. We revised in the manuscript*

* The phrase "lifestyle modification practices" is vague. Specify the practices under discussion.

***Response:*** *Thank you for your valuable comment. We revised in the manuscript*

* "All testing negative for malaria", suggestion, "All of which tested negative for malaria."

***Response:*** *Thank you for your valuable comment. We revised in the manuscript*

* "Task forces and rapid response teams were established to investigate the outbreak's extent and implement control measures.", suggestion, Consider specifying the composition or role of these teams for added clarity.

***Response:*** *Thank you for your valuable suggestion. We have revised as”* Rapid Response Teams (RRT) consisted a surveillance officer, clinician, laboratory technician, and risk communication officer who work together to tackle outbreaks. Their responsibilities include investigating the outbreaks, assisting local health personnel in implementing control measures, and evaluating the effectiveness of strategies. A taskforce, formed with local authorities and community leaders, aims to raise awareness and mobilize resources. This approach ensures that responses are coordinated and tailored, leading to more effective management of dengue outbreaks” *in the manuscript*

* "According to the EPi curve", suggestion, "According to the epidemic (EPi) curve." (Always expand abbreviations on first use.)

***Response:*** *Thank you for your valuable suggestion. We have revised as”*According to the epidemic (EPi) curve, the outbreak began on July 25, 2023, marked by the index case” *in the manuscript*

* "The curve indicated that the potential initial exposure date could have occurred...", suggestion, Rephrase to: "The curve suggested that initial exposure likely occurred..."

***Response:*** *Thank you for your valuable comment. We revised in the manuscript*

* "The presence of the Aedes aegypti mosquito species in the study area was confirmed using standard morphological identification techniques[22]", suggestion, Add a period after the citation number.

***Response:*** *Thank you for your valuable comment. We revised in the manuscript*

* "During their visits to 1,789 households, the team identified 2,219 water-holding containers that had the potential to serve as mosquito breeding sites.", suggestion, Simplify: "...identified 2,219 potential mosquito breeding sites in 1,789 households."

***Response:*** *Thank you for your valuable comment. We revised in the manuscript*

* "This result indicates that a substantial majority of the participants, specifically 128(85.3%) did not have close contact...", suggestion, Add a comma after 128(85.3%) for clarity.

***Response:*** *Thank you for your valuable comment. We revised in the manuscript*

* Ensure consistent formatting of percentages and numbers across the text (e.g., "128(85.3%)", suggestion, "128 (85.3%).").

***Response:*** *Thank you for your valuable comment. We revised in the manuscript*

* Include context for why close contact and educational status are relevant to the study outcomes.

***Response:*** *Thank you for your valuable comment.* *We revised our introduction part by adding* one *paragraph as* “Close contact play a crucial role in study outcomes since being near dengue-infected individuals increases the risk of transmission through mosquito bites. Asymptomatic infections often occur among those in close community ties, highlighting the importance of understanding how transmission dynamics work within these groups. Educational status significantly influences individuals' understanding and practices of dengue prevention. Higher education levels correlate with better knowledge of preventive measures like using Long-Lasting Insecticide Nets (LLINs) and removing mosquito breeding sites. Well-informed individuals are more likely to adopt effective prevention strategies” *in the manuscript*

* "Knowledge regarding the biting times of Aedes aegypti mosquitoes was limited...", suggestion, Avoid repetition of "knowledge" in consecutive sentences.

***Response:*** *Thank you for your valuable comment. We revised in the manuscript*

* "Individuals who did not use Long-Lasting Insecticide Nets (LLINs) (LLINs)...", suggestion, Remove duplicate reference to LLINs.

***Response:*** *Thank you for your valuable comment. We revised in the manuscript*

* "A dengue outbreak has been officially declared in Metahara town.", suggestion, Should be past tense: "was officially declared."

***Response:*** *Thank you for your valuable comment. We revised in the manuscript*

* "Among 150 febrile cases identified through active case searches sent to health centers, 54 were confirmed as dengue cases.", suggestion, Clarify: "Of the 150 febrile cases identified and referred to health centers, 54 were confirmed as dengue cases."

***Response:*** *Thank you for your valuable comment. We revised in the manuscript*

* "This targeted approach aims to mitigate mosquito populations...", suggestion, Use past tense: "This targeted approach aimed to..."

***Response:*** *Thank you for your valuable comment. We revised in the manuscript*

* "The Oromia region is currently facing multiple outbreaks, including cholera and malaria, alongside a significant lack of trained personnel knowledgeable about arboviral diseases.", suggestion, "...cholera and malaria, coupled with a shortage of trained personnel in arboviral diseases."

***Response:*** *Thank you for your valuable comment. We revised in the manuscript*

Discussion
* "Dengue has emerged as a significant public health concern in Ethiopia, characterized by underreported epidemiology due to inadequate arboviral surveillance, challenges in case confirmation, and a lack of isolation in endemic areas."
Issues: "Underreported epidemiology" is unclear and awkward.

***Response:*** *Thank you for your valuable comment. We revised in the manuscript*

* The phrase "a lack of isolation in endemic areas" could be clarified.
Suggestion: "Dengue has emerged as a significant public health concern in Ethiopia due to inadequate arboviral surveillance, challenges in case confirmation, and limited isolation measures in endemic areas."

***Response:*** *Thank you for your valuable comment. We revised in the manuscript*

* "Understanding the current burden of dengue and identifying major risk factors are essential for mitigating future outbreaks of dengue and other arboviral diseases in Ethiopia through appropriate recommendations."
Issues: The sentence is lengthy and can be simplified.
Suggestion: "Understanding the current burden of dengue and its risk factors is crucial for preventing future outbreaks and other arboviral diseases in Ethiopia."

***Response:*** *Thank you for your valuable comment.* Understanding the current burden of dengue and its risk factors is crucial for preventing future outbreaks and other arboviral diseases in Ethiopia” *in the manuscript*

* "Prior to this, the only laboratory-confirmed dengue outbreak in Ethiopia occurred in Dire Dawa city in 2013."
Issues: "City" is redundant as Dire Dawa is commonly recognized as a city.
Suggestion: "Prior to this, the only laboratory-confirmed dengue outbreak in Ethiopia was in Dire Dawa in 2013."

***Response:*** *Thank you for your valuable comment.* We have revised as “Prior to this, the only laboratory-confirmed dengue outbreak in Ethiopia was in Dire Dawa in 2013” *in the manuscript*

* "The findings of this study reveal that all patients diagnosed with dengue exhibited fever and headache, with both symptoms occurring in 100% of cases among individuals presenting with dengue-related clinical manifestations."
Issues: "With both symptoms occurring in 100% of cases" is repetitive.
Suggestion: "All dengue patients in this study exhibited fever and headache, consistent with prior findings from Dire Dawa."

***Response:*** *Thank you for your valuable comment.* All dengue patients in this study exhibited fever and headache, consistent with prior findings from Dire Dawa” *in the manuscript*

* Surveillance gaps and a lack of community awareness are mentioned multiple times. Consider consolidating these themes for better readability.
Suggestion: Combine mentions of the need for improved surveillance into one cohesive paragraph.

***Response:*** *Thank you for your valuable comment. We revised in the manuscript*

* When comparing case fatality rates across regions, the discussion could benefit from clearer transitions and summarization:
Suggestion: "Case fatality rates varied across regions, with Ethiopia's rate of 0.88% aligning with Bhutan and Nepal (~1%) but significantly lower than India, Indonesia, and Myanmar (3-5%). These differences highlight geographical and systemic variations in dengue management."

***Response:*** *Thank you for your valuable comment. We revised in the manuscript*

* "The absence of longitudinal data restricts the ability to assess temporal trends in dengue incidence or evaluate the long-term effectiveness of implemented control measures."
Issue: Sentence is correct but slightly long.
Suggestion: "The lack of longitudinal data limits the assessment of dengue trends and the evaluation of long-term control measures."

***Response:*** *Thank you for your valuable comment. We revised in the manuscript*

* "Understanding the degree of community involvement is crucial for evaluating the success of educational campaigns aimed at reducing dengue transmission."
Issue: Could be more concise.
Suggestion: "Community involvement is key to evaluating the success of educational campaigns against dengue."

***Response:*** *Thank you for your valuable comment. We revised in the manuscript*

* "The ongoing circulation of dengue serotype DENV-3 in Metahara is particularly concerning; as this serotype is associated with severe clinical manifestations, including dengue hemorrhagic fever (DHF)."
Issue: Misuse of semicolon.
Suggestion: "The ongoing circulation of dengue serotype DENV-3 in Metahara is particularly concerning, as it is associated with severe clinical manifestations, including dengue hemorrhagic fever (DHF)."

***Response:*** *Thank you for your valuable comment. We revised in the manuscript*

* "Many residents lack essential information about dengue virus transmission and prevention strategies, which diminishes their ability to protect them effectively."
Issue: "Protect them" is awkward.
Suggestion: "Many residents lack essential information about dengue transmission and prevention, reducing their ability to protect themselves."

***Response:*** *Thank you for your valuable comment. We revised in the manuscript*

* The conclusion could explicitly link study findings to actionable recommendations. For example:
"Public health authorities must prioritize implementing Integrated Vector Management and community education to address key risk factors identified in this study."

***Response:*** *Thank you for your valuable comment. We revised in the manuscript*

**Journal Requirements:**

1) We note that your Data Availability Statement is currently as follows: "Data is available at the hand of principal investigator and can access if required". Please confirm at this time whether or not your submission contains all raw data required to replicate the results of your study. Authors must share the “minimal data set” for their submission. PLOS defines the minimal data set to consist of the data required to replicate all study findings reported in the article, as well as related metadata and methods (<https://journals.plos.org/plosone/s/data-availability#loc-minimal-data-set-definition>).
For example, authors should submit the following data:
- The values behind the means, standard deviations and other measures reported;
- The values used to build graphs;
- The points extracted from images for analysis..
Authors do not need to submit their entire data set if only a portion of the data was used in the reported study.
If your submission does not contain these data, please either upload them as Supporting Information files or deposit them to a stable, public repository and provide us with the relevant URLs, DOIs, or accession numbers. For a list of recommended repositories, please see <https://journals.plos.org/plosone/s/recommended-repositories>.
If there are ethical or legal restrictions on sharing a de-identified data set, please explain them in detail (e.g., data contain potentially sensitive information, data are owned by a third-party organization, etc.) and who has imposed them (e.g., an ethics committee). Please also provide contact information for a data access committee, ethics committee, or other institutional body to which data requests may be sent. If data are owned by a third party, please indicate how others may request data access.

***Responses:*** Thank you for your feedback and requests for clarification. We acknowledge your point regarding the availability of data for replication of the study. To clarify, when we previously stated that "there is data at the hand of the Authors," we did not intend to imply that we had omitted any data. Instead, we meant to convey that we have provided all relevant data to the journal as part of our submission and also we have it at our hand. We understand the importance of data transparency and replication in scientific research, and we assure you that all necessary data supporting our findings has been included in our submission. If further details or specific datasets are required for review or replication purposes, please let us know, and we will be happy to provide additional information.

Thank you for your understanding as we work to ensure clarity in our communication.

**Reviewers' comments:**

Reviewer's Responses to Questions

**Key Review Criteria Required for Acceptance?**

As you describe the new analyses required for acceptance, please consider the following:


**Methods**


-Are the objectives of the study clearly articulated with a clear testable hypothesis stated?

-Is the study design appropriate to address the stated objectives?

-Is the population clearly described and appropriate for the hypothesis being tested?

-Is the sample size sufficient to ensure adequate power to address the hypothesis being tested?

-Were correct statistical analysis used to support conclusions?

-Are there concerns about ethical or regulatory requirements being met?

Reviewer #2: This is the third revision. The authors have addressed the comments.

**Responses**: Thank you for your acknowledgement

**Results**


-Does the analysis presented match the analysis plan?

-Are the results clearly and completely presented?

-Are the figures (Tables, Images) of sufficient quality for clarity?

Reviewer #2: This is the third revision. The authors have addressed the comments.

**Responses**: Thank you for your acknowledgement

**Conclusions**


-Are the conclusions supported by the data presented?

-Are the limitations of analysis clearly described?

-Do the authors discuss how these data can be helpful to advance our understanding of the topic under study?

-Is public health relevance addressed?

Reviewer #2: This is the third revision. The authors have addressed the comments.

**Responses**: Thank you for your acknowledgement

**Editorial and Data Presentation Modifications?**


Use this section for editorial suggestions as well as relatively minor modifications of existing data that would enhance clarity. If the only modifications needed are minor and/or editorial, you may wish to recommend “Minor Revision” or “Accept”.

Reviewer #2: This is the third revision. The authors have addressed the comments.

**Responses**: Thank you for your acknowledgement.

**Summary and General Comments**


Use this section to provide overall comments, discuss strengths/weaknesses of the study, novelty, significance, general execution and scholarship. You may also include additional comments for the author, including concerns about dual publication, research ethics, or publication ethics. If requesting major revision, please articulate the new experiments that are needed.

Reviewer #2: This is the third revision. The authors have addressed the comments.

**Responses**: Thank you for your acknowledgement.

PLOS authors have the option to publish the peer review history of their article ([what does this mean?](https://track.editorialmanager.com/CL0/https:%2F%2Fjournals.plos.org%2Fplosntds%2Fs%2Feditorial-and-peer-review-process%23loc-peer-review-history/1/010f01944c77e270-8baf5dc6-641e-4e3a-84a3-7208d465be8e-000000/SDXFu3-12dwqjkjNcDG3njv9ej8VU6lBBlPYF4wz0ZM=192)). If published, this will include your full peer review and any attached files.


If you choose “no”, your identity will remain anonymous but your review may still be made public.


**Do you want your identity to be public for this peer review?** For information about this choice, including consent withdrawal, please see our [Privacy Policy](https://track.editorialmanager.com/CL0/https:%2F%2Fwww.plos.org%2Fprivacy-policy/1/010f01944c77e270-8baf5dc6-641e-4e3a-84a3-7208d465be8e-000000/RUTGRBjggwiJo80asLirW-Fj8nj__56kRl2TDL-Hqoc=192).

Reviewer #2: **Yes:** Ruth Aralí Martínez Vega

**Responses**: Thank you

[NOTE: If reviewer comments were submitted as an attachment file, they will be attached to this email and accessible via the submission site. Please log into your account, locate the manuscript record, and check for the action link "View Attachments". If this link does not appear, there are no attachment files.]

**Figure resubmission:**

While revising your submission, please upload your figure files to the Preflight Analysis and Conversion Engine (PACE) digital diagnostic tool, <https://pacev2.apexcovantage.com/>. PACE helps ensure that figures meet PLOS requirements. To use PACE, you must first register as a user. Registration is free. Then, login and navigate to the UPLOAD tab, where you will find detailed instructions on how to use the tool. If you encounter any issues or have any questions when using PACE, please email PLOS at [figures@plos.org](mailto:figures@plos.org). Please note that Supporting Information files do not need this step. If there are other versions of figure files still present in your submission file inventory at resubmission, please replace them with the PACE-processed versions.

**Reproducibility:**

To enhance the reproducibility of your results, we recommend that authors of applicable studies deposit laboratory protocols in [protocols.io](http://protocols.io/), where a protocol can be assigned its own identifier (DOI) such that it can be cited independently in the future. Additionally, PLOS ONE offers an option to publish peer-reviewed clinical study protocols. Read more information on sharing protocols at <https://plos.org/protocols?utm_medium=editorial-email&utm_source=authorletters&utm_campaign=protocols>
